# Community-directed educational intervention for malaria elimination in Bhutan: quasi-experimental study in malaria endemic areas of Sarpang district

**DOI:** 10.1186/1475-2875-12-132

**Published:** 2013-04-17

**Authors:** Tashi Tobgay, Deki Pem, Ugyen Dophu, Shyam P Dumre, Kesara Na-Bangchang, Cristina E Torres

**Affiliations:** 1 Ministry of Health, Thimphu, Bhutan; 2 Royal Institute of Health Sciences, Thimphu, Bhutan; 3 Thammasat University, Rangsit Campus, Bangkok, Thailand; 4 Thailand Centre of Excellence on Drug discovery and Development, Thammasat University, Bangkok, Thailand; 5 Forum for Ethical Review Committee in the Asian & Western Pacific Region, Thammasat University, Bangkok, Thailand

## Abstract

**Background:**

As per the World Malaria Report 2011, there was a 17% reduction in morbidity and 26% reduction in mortality in 2010, compared to 2000. In Bhutan, there were only 194 malaria cases in 2011 as compared to 5,935 cases in 2000. As the country moves towards an elimination phase, educating the community and empowering them on malaria prevention and control is imperative. Hence, this study was conducted to elucidate the effectiveness of the community-directed educational intervention on malaria prevention and control in malaria-endemic areas of Sarpang district, Bhutan.

**Methods:**

This quasi-experimental study design was conducted using both qualitative and quantitative data collection methods. In-depth interviews and focus group discussions were carried out in addition to household survey using a structured questionnaire conducted before and after the intervention. Intervention was conducted using community action groups, who were provided with training and which then developed action plans for implementation of interventions within their communities.

**Results:**

The study resulted in a significant improvement in knowledge and attitude in intervention as compared to control during the post-intervention survey (p < 0.001). The practice score was higher in the control group both during pre- and post-intervention, however, the mean ( ±sd) score of practice in intervention group increased from 6.84 ± 1.26 in pre-intervention to 8.35 ± 1.14 in post-intervention (p < 0.001), where as it decreased from 9.19 ± 1.78 to 9.10 ± 1.98 in the control group (p = 0. 68). When comparing pre- and post- in the intervention group, there was significant improvement during post-intervention in knowledge, attitude and practice (p < 0.001).

**Conclusions:**

The findings from this study corroborate that community-directed interventions can be utilized as an effective means for improving knowledge, attitude and practice in the malaria-endemic areas of Bhutan. Further studies are needed to see the long-term effect and sustainability of such interventions.

## Background

Currently, with the availability of the best interventions, including long-lasting insecticidal nets (LLINs) and artemisinin combination therapy (ACT), morbidity and mortality due to malaria is an unnecessary cause. Yet malaria affects 106 countries and, according to the 2011 World Malaria Report, there were 216 million malaria episodes, of which 86% of cases were in children under five years of age, and 655,0000 malaria deaths in 2010. Globally, despite a 17% reduction in the number of reported cases and 26% reduction in the number of deaths in 2010 as compared to 2000, malaria is still a ubiquitous killer [1]. Although, Africa is known to have the most high-risk areas, 4.3 million reported cases and 2,426 deaths in 2010, were from Southeast Asia. Further, some of these countries, namely Bangladesh, Myanmar and Timor-Leste, showed an increasing trend, unlike other countries. In addition, frequent outbreaks, high *Plasmodium falciparum* cases, and resistance to drugs and chemicals means malaria situations in Asia are unstable and volatile [1,2].

Among the WHO Regional Office Southeast Asia (WHO-SEARO) countries, Bhutan is successful in terms of malaria prevention and control with considerable reduction in morbidity and mortality. The number of cases reduced from 5,935 cases in 2000 to just 194 cases in 2011. Similarly, there was only one death in 2011 as compared to 15 deaths in 2000 [3]. Although, absolute numbers are small, this represents a substantial challenge for a small country of only 634,982 people and with more than half its population at risk of getting malaria. Moreover, malaria in Bhutan is dynamic and unstable which is evident from an upsurge of reported cases in 2009 to 972 cases with five deaths, almost a triple-fold increase from 329 cases, with two deaths in 2008. Bhutan shares a border with the malaria-endemic Indian states of Assam and West Bengal and with unfettered movement of people across these borders posing a tremendous challenge for malaria prevention and control [4].

For maximal benefit from the current effective malaria prevention and control armamentariums, such as LLINs or indoor residual spraying (IRS), early diagnosis with microscopic examination and rapid diagnosis tests and treatment of confirmed malaria cases with ACT, it is imperative that communities are educated to enable them to make informed decisions [5,6]. However, different societies hold a variety of beliefs according to their culture, education, religion and economic status. These local contexts measured through knowledge, attitude and practice (KAP) surveys conducted in many countries showed that communities linked malaria causalities to various agents and actions such as mosquito bites, sitting in the sun, eating unripe fruits, drinking dirty water or house flies, and severe malaria with convulsions relate to witch crafts and evil spirits [7-15]. Similarly, in Bhutan, misconceptions about malaria persist with some people believing that malaria is caused by staying long hours in sun, by local spirits, and taking fruits with sour taste [16]. Such beliefs could lead to delayed treatment and non-utilization of effective interventions. These misconceptions of malaria prevention and control can be negated through effective community participatory education [17-19]. Since the Declaration of Alma-Ata, community participation is in the forefront of primary health care and is considered a necessary imperative for sustainable interventions [20,21]. The success of any public health interventions anchors on the availability of effective interventions, its accessibility to the people in need, and effective utilization by the communities at risk of malaria [22]. This requires community mobilization, participation and empowerment for the interventions to be sustainable, equitable, accessible and beneficial to communities in need [23].

Prevention and control of malaria whether use of LLINs, IRS, environmental management to prevent breeding sites or promoting early diagnosis and treatment, requires constant involvement of community. However, despite receiving major focus, the issues of community participation has been rhetorical and lacked a clear path on the process of empowering communities in addressing the health needs of their communities. Community-directed interventions (CDI) provide a focused approach to involve communities in decisions and in taking responsibility for the betterment of their health [24]. CDI was a successful strategy in Africa for the management of onchocerciasis by the African Programme for Onchocerciasis Control (APOC) [25]. Such a community-directed approach has the dual benefit of community empowerment and health system support and it can accelerate health promotion, disease prevention, control and elimination which are critical for the achievement of the Millennium Development Goals (MDGs) [26].

The heath system in Bhutan has undergone a decentralization process with strong emphasis on primary health care. This resulted in elimination of some diseases, such as leprosy, and reduction in the burden of infectious diseases and the achievement of some of the MDGs’ targets. Bhutan has a functional primary health care system with basic health units (BHUs) and village health worker (VHWs) within the community. With Bhutan’s transition in 2008 to a parliamentary government, community systems have been further strengthened by the establishment of local government systems at village and at the sub-district level. This provides an opportunity to strengthen community involvement in developing interventions for malaria prevention and control. There is fertile ground for the use of CDI in implementing various malaria strategies for sustainable elimination from Bhutan.

This study used the principles of CDI though community action groups that are elected by the community, endorsed by a local leader and BHU, to implement action plans, one of which is community education.

## Methods

**Figure 1:**
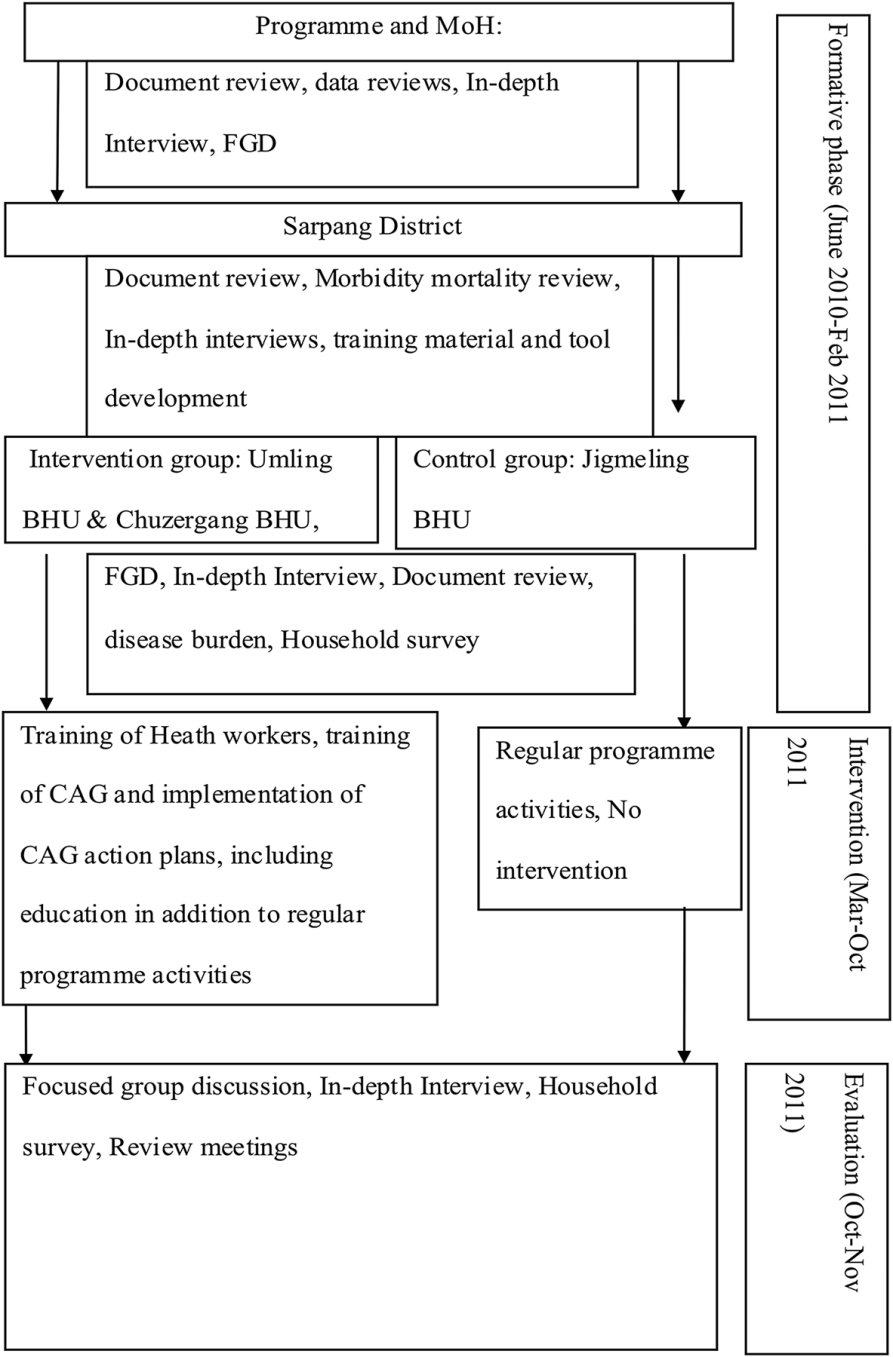
Schematic diagram for research activities.

This is a quasi-experimental study design using triangulation of qualitative and quantitative data collection methods. The implementation of the community-directed educational intervention (CDEI) involved training health staff and local leaders who in turn trained community action groups (CAGs) that were nominated by their communities (Figure 1). The training modules were developed by principal investigator in discussion with basic health unit staff and topics included malaria transmission, care and use of LLINs, proper use of IRS, control of mosquito breeding sites, importance of early diagnosis and treatment. The respective BHU staff trained the CAG members using the pre developed training module. As part of their activity, the CAG members conducted monthly cleaning campaigns during which they also provided educational session on malaria prevention and control. A flip chart was also developed on malaria prevention and control to be used by CAG while imparting education to the community. The CAGs developed action plans for malaria prevention and control that included conducting educational sessions in their local villages, organizing cleaning campaigns to reduce mosquito breeding sites and monitoring mosquito net use. BHU staff and local leaders monitored the implementation of these activities. CAGs presented their progress reports during review meetings in the presence of local leader and BHU staff.

For qualitative data, 13 in-depth interviews and 12 focus group discussions were conducted before intervention and nine in-depth interviews and nine focus group discussions after intervention. The quantitative data included interviewing 280 households per group before intervention and after intervention with a total of 560 households interviewed. Houses that were available for interview, household members above 18 years of age if the head of the household was not available, is currently resident of that community, provided informed consent were included in the survey and household that were locked and no competent interviewee even after trying three times and those who did not consent were excluded from the survey.

**Figure 2:**
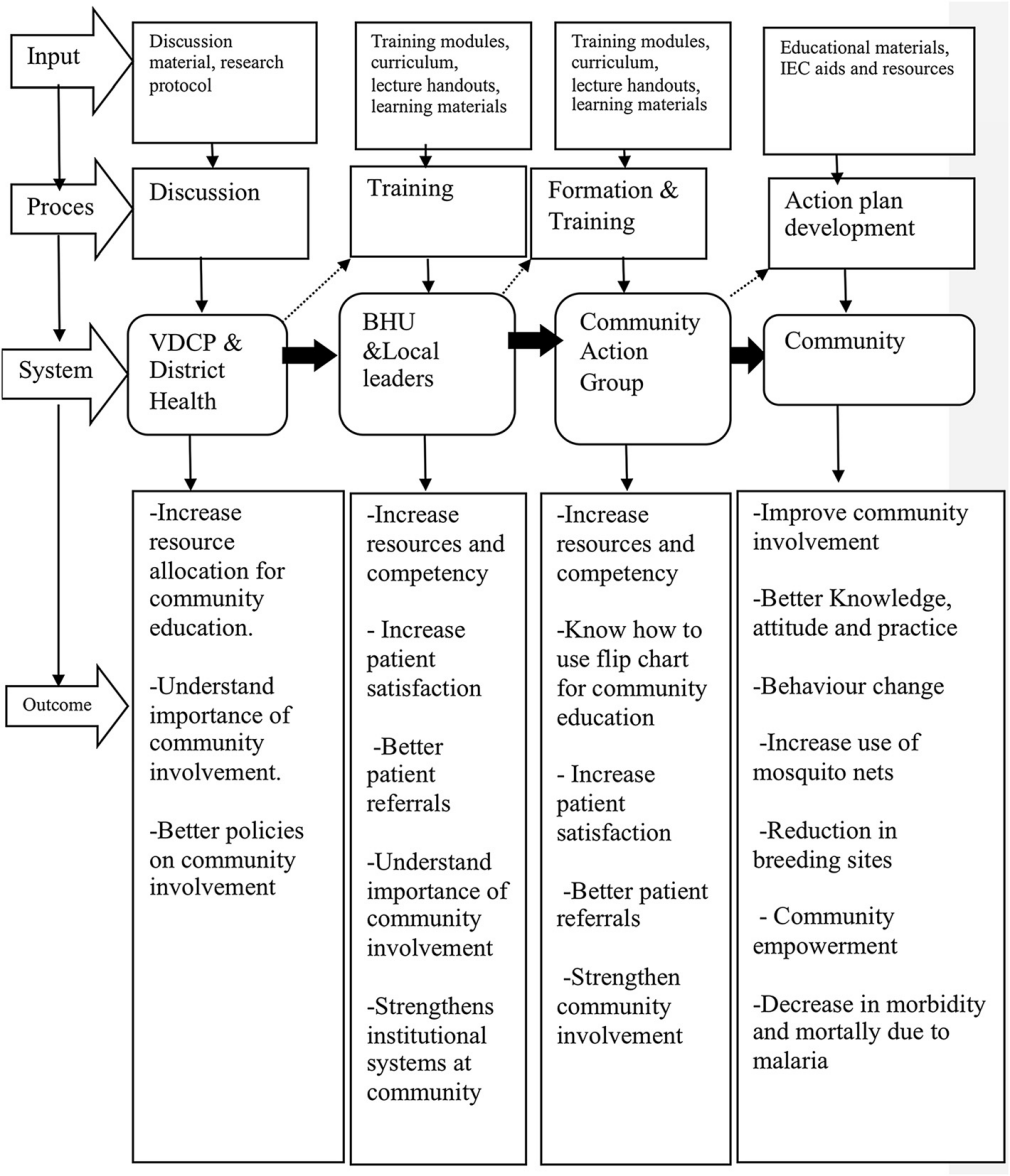
Logical process of the community-directed educational intervention.

The intervention included formation of the CAG within the community, training of the CAG by the health workers and local leaders, development of action plans by the CAG and implementation of these action plans (Figure 2).

Both qualitative data and quantitative data were collected before the intervention and six months after the intervention.

### Sampling and data analysis

Sarpang was purposely selected from seven malaria-endemic districts in Bhutan based on the highest malaria cases over past years with malaria incidence of 4.05 cases per 1000 population in 2010 (Table 1). The BHUs were selected based on highest number of malaria cases having more than 6.19 malaria cases per 1000 population. Two BHUs, namely Chuzergang BHU and Umling BHU, were selected for intervention and Jigmeling BHU was selected as a control area based on population size and the proximity between each other. Since the focus was on rural areas, hospitals were not included. The intervention and control group were selected to provide similar baseline in terms of malaria cases, population demographics and to limit contamination. Systematic random sampling was used to select households for interview for each sampling. This research was approved by the Bhutan Research Ethics Board of Health and also by World Health Organization-Research Ethics Review Committee (Protocol ID A80328 dated 27-7-2010).

**Table 1:** Demographic details of control and intervention

**Demographics**	**Intervention**		**Control**	**District**	**Nation**
	**Chuzergang**	**Umling**	**Jigmeling**	**Sarpang**	**Bhutan**
Population	2909	1451	4233	61,434	634,982
Households	632	315	920	13,355	138,039
VHW	3	8	6	52	1200
Blood slide collection (2010)	807	782	1807	21478	54616
Malaria cases (2010)	18	18	23	249	436

The principal investigator conducted the in-depth interview and focus group discussions along with co-investigator who noted the minutes in English and also recorded the interview-using recorder. This field notes were translated, reviewed, extended and converted to Microsoft Word by the principle investigator after tallying with the audio records. The reviewed notes and write-ups were grouped under the different themes of common illness within communities, prevention activities including use of LLIN and IRS, treatment and health seeking behaviour, educational activities conducted within communities as per the interview guide which was used during the interview session.

The quantitative data was obtained using structured household survey. For data collection of household survey, local non-formal teachers were recruited. They were provided two days training by principal investigator on questionnaire content, data collection methodology, communication skills, obtaining informed consent and filling up the questionnaire. The questionnaire were developed in English and then translated to Dzongkha, which is the national language of Bhutan. The enumerators administered either in Dzongkha or English depending upon the language skills of the respondent. The enumerators also physically counted the mosquito nets and checked on the condition of the nets whether there were holes or not and if nets were useable. The questionnaire was piloted in a community which is away from the study area but had similar ethnic, socio-economic and demographic mixture.

For analysis, the data entry format was developed in EpiData, version 3.1 using skip, legal and range values to reduce errors. Data was entered into this format and exported to Microsoft Office Excel 2007 for manual cleaning and checking and further exported to SPSS version 12.0 software (SPSS Co Ltd, Thailand) for analysis. *T*-test of independent samples was performed to compare before and after intervention and also control *versus* intervention, at a statistical significance level of α = 0.05.

## Results

### Qualitative

The common illnesses in communities as stated by the interviewees were cough and cold, fever, eye problem and diarrhoea. Other diseases mentioned by the communities were tuberculosis, typhoid fever, malaria, diabetes and blood pressure. The health workers of BHUs said that fever, acute respiratory illness, common cold and diarrhoea are the most common illness in the community, although, an interview with the referral hospital and the ministry revealed that non-communicable diseases such as blood pressure and diabetes are becoming more common in recent years.

Most of the participants stated that for most illness, people go to health facility as soon as possible due to fear of some dangerous diseases. They said that for fever, most people go to health facility due to fear for malaria. One of the participants said “*the treatment from the health facility is the only remedy for malaria and no other treatment including local healers will cure and therefore, people go to health facility for treatment*”. However, consulting and going to local healers before going to hospitals are not uncommon. One of the local healers also stated that people usually come to him to seek guidance and advise on the course of treatment. He said “*for some illnesses like the one possessed by local spirits would be very dangerous to go to health facility and there are chances of death if the patient receives injections for such conditions”*.

Malaria is known by various names depending upon their dialects namely “Tshenay” in Dzongkha, “Tshepai nat” in Sharchop and “Joru” in Lhotsham. Most of the participants said that malaria is caused by not keeping the surroundings clean which promotes mosquito breeding. Other causes stated by the participants were; working hard under the sun and the rain, cooking food with firewood, dirty surrounding, people sleeping outside during harvest season, unhygienic house, drinking unclean water or cold water in hot weather which disrupts body system and taking sour things. The health workers believe that people get malaria when they are not sleeping under mosquito nets especially while they are guarding their crops or some people get drunk and sleep outside. The participants confirmed that currently the government has distributed mosquito nets for all people depending upon the family size. However, most participants did not know how to take care of the mosquito nets such as when, how and how many times to wash the mosquito nets except some participants in the post intervention group. The participants said that some of the malaria symptoms were fever, body ache, joint pains, and chills. Most communities believed that malaria can only be diagnosed through blood test at health facilities and can be cured only by taking medicines provided through health facilities. However, some participants expressed that often, they have to do multiple blood test to confirm the diagnosis of malaria.

The participants said that most people in their community were aware of malaria as a result of frequent education and awareness campaigns conducted by health workers. They stated that the health workers provide educational sessions at least once or twice a year although those who have attended such sessions could not recollect meeting timings or the topics covered and usually health workers combine many health topics including HIV, TB and malaria during the session. However, during the post intervention interview, in the intervention area, the participants stated the community action groups organize cleaning campaigns twice a month on auspicious days, when the village people take rest from the plantation, and during such session CAG members provided malaria education session at the beginning of the cleaning campaign using a flip chart. The communities stated that such initiatives by the communities are good as it is for the betterment of their communities. Further they commented that such systems are good as the communities have nominated their CAG, endorsed by the community leaders and also monitored by their elected local leaders which provides them ownership for taking care of their health within their communities.

### Quantitative

Overall, respondents consisted of 62% female, 58% having no formal education, 72% farmers, 49% heads of household, 72% Buddhist, 27% Hindu, and a mean age of 43 ± 14 years (Table 2). The two major ethnic groups within the study were 30% Sharchops (from eastern Bhutan) and 34% Lhotsham (of southern Bhutan with Nepali origin). The mean overall household size was 4.9 ± 2.3. Amongst the households having mosquito nets, 97% had at least one net in good condition with mean of 2.9 ± 1.5 good condition LLINs. Some 93% of the respondents said that they slept under a mosquito net the previous night. The reasons stated for not sleeping under mosquito nets were insufficient nets at the house, nets taken by children to their boarding school, feeling hot while sleeping under mosquito nets. Within the previous 12 months, 96% of the respondents reported that they had had their houses sprayed with IRS. There were more respondents in the post intervention group (p < 0.001) who had heard about malaria prevention and control from CAGs as compared to the control group, and also when compared with pre-intervention group (p < 0.001) (Table 3). There were more health education sessions conducted in the post-intervention group compared to the control (p < 0.001). This was an important outcome as the interventions were expected to enable CAG to provide education on malaria within their communities and further sustain these activities. Of those who rated useful or very useful, there was a significantly higher number in the intervention group both during pre- (p = 0.002) and post-(p < 0.001) intervention surveys when compared to control group. The mean score for KAP increased in the intervention group as compared to pre-intervention group (Table 4). In the control group, despite an increase in mean score for knowledge, the mean score for attitude and practice decreased during post-intervention as compared to pre-intervention.

**Table 2:** Socio-demographic profile of households

**Characteristics**	**Pre-intervention**		**Post-intervention**	
	**Control (n = 140)**	**Intervention (n = 140)**	**Control (n = 140)**	**Intervention (n = 140)**
Female (n,%)	85 (60.7)	101 (72.1)	63 (45.0)	96 (68.6)
No formal education (n,%)	84 (60)	70 (50.0)	79 (56.4)	91 (65)
Farmer (n,%)	117 (83.6)	128 (91.4)	112 (86.4)	130 (95.0)
Married (n,%)	127 (90.7)	119 (85.0)	125 (89.3)	120 (85.7)
Road network connections (n,%)	122 (87.1)	106 (75.7)	123 (87.9)	123 (87.9)
Electricity connections (n,%)	134 (95.7)	138 (98.6)	134 (95.7)	133 (95.0)
Mobile phone availability (n,%)	126 (90.0)	120 (85.7)	114 (81.4)	119 (85)
No transportation facilities (n,%)	89 (63.6)	113 (80.7)	86 (61.4)	121 (86.4)
Age of the respondents (mean ± SD)				43.5 ± 14.7	42.8 ± 15.3	43.1 ± 13.5	44.1 ± 14.7
Number of people currently staying/household (mean ± SD)				5.5 ± 3.05	4.72 ± 1.98	4.93 ± 2.12	4.39 ± 1.83
Number of good condition mosquito nets (mean ± SD)				3.0 ± 1.7	3.1 ± 1.7	3.0 ± 1.4	2.6 ± 1.2

**Table 3:** Comparison between control and of malaria prevention and control indicators

**Characteristics**	**Pre-intervention**			**Post-intervention**			**Pre-/Post-*p*-value**	
	**Control n (%)**	**Intervention n (%)**	***p-*value**	**Control n (%)**	**Intervention n (%)**	***p-*value**	**Control**	**Intervention**
Slept under net the previous night	133 (95.0)	136 (97.1)	0.356	124 (89.9)	128 (91.4)	0.652	0.105	0.039^*^
Houses sprayed within past 12 months	138 (98.6)	138 (98.6)	0.159	122 (87.1)	137 (97.9)	0.120	0.010^*^	0.994^*^
Heard about malaria from health staff	125 (89.3)	124 (88.6)	0.840	75 (57.7)	121 (89.6)	<0.001^*^	<0.001^*^	0.778
Heard about malaria from CAG	18 (12.9)	14 (10)	0.452	13 (10)	66.7	<0.001^*^	0.462	<0.001^*^
Heard about malaria from radio	62 (44.3)	52 (37.1)	0.224	52 (40.0)	78 (57.8)	0.004^*^	0.476	0.001^*^
Regularly attended health education sessions	72 (77.4)	32 (58.2)	0.013^*^	52 (54.2)	62 (47)	0.297	0.001^*^	0.192
Never attended education sessions	1 (1.1)	8 (14.5)	0.001^*^	4 (4.2)	8 (6.1)	0.522	0.182	0.063
Malaria covered during health education	89 (63.6)	43 (30.7)	<0.001^*^	88 (91.7)	109 (82.6)	0.048^*^	<0.001^*^	<0.001^*^
Average number sessions past six months	2.1 ± 0.96	2.13 ± 1.31	0.839	1.58 ± 0.66	2.62 ± 1.29	<0.001^**^	<0.001^**^	0.019^**^

^*^Statistically significant for *χ*2 test of independence at 0.05 significance level.

^**^ Statistically significant for independent *T* test at 0.05 significance level

**Table 4:** Comparison between knowledge, attitude and practice outcome

**Variables**	**Pre-intervention**			**Post-intervention**			**Pre-/Post-*p-*value**	
	**Control.**	**Intervention**	***p-*value**	**Control**	**Intervention**	***p-*value**	**Control**	**Intervention**
Mean score “knowledge” (sd)	10.29 ± 1.66	10.36 ± 1.95	0.742	11.13 ± 1.9	12.64 ± 2.16	<0.001^**^	<0.001^**^	<0.001^**^
Mean score “optimism” (sd)	11.66 ± 1.86	11.81 ± 1.72	0.464	11.59 ± 2.22	12.53 ± 1.4	<0.001^**^	0.774	<0.001^**^
Mean score “good practice” (sd)	9.19 ± 1.78	6.84 ± 1.26	<0.001^**^	9.10 ± 1.98	8.35 ± 1.14	<0.001^**^	0.680	<0.001^**^

^**^ Statistically significant for independent *t*-test at a=0.05.

## Discussion

This study was conducted in a rural setting of Sarpang district, which is endemic to malaria and other vector-borne diseases. The majority of respondents were farmers and illiterate with over 60% of respondents not having any transport facilities, indicating a high prevalence of abject poverty in the study area. In Bhutan, 23% of the population belong to households whose per capital consumption is below the total food poverty line of Nu688.96 (US$15) per person per Month; most belong to rural communities [27]. The average household size of 4.9 persons per household in this study was consistent with the national figure of 4.6 [28]. Malaria affects poor and rural communities and these communities often live in a vicious cycle of poverty and disease [29-31]. A study conducted in South Africa showed that people living in traditional houses, made of earth and wood are at higher risk of getting malaria than people living in western-style houses [32].

The LLIN is considered the single most effective means of malaria prevention and control [33,34]. Bhutan began distribution of LLINs in 2006 and since then LLINs have been distributed regularly according to family size with the objective to achieve universal coverage. Except for two households, which had no LLINs, the average number of good condition LLIN per household was 2.9 good condition nets. Considering the household size of 4.6 there was at least one net per 1.6 people indicating universal net coverage [35]. These findings were similar to an malaria indicator survey which found that 92% of the respondents had at least one LLIN, with an average of 2.8 bed nets per household [36]. This study showed that the mean number of good condition mosquito nets was 3.02 ± 1.7 in pre-intervention and 2.78 ± 1.3 in post-intervention. Therefore, on average, nets tend to wear out at the rate of 0.24 ((3.02-2.78) = 0.24*100 = 24%) per year. This indicates that despite claims that LLINs would be effective at least three years in a community setting, the net wither rate is not uniform in all households [37]. There is a need to monitor the net availability at households regularly and distribute accordingly. Further, communities lacked specific knowledge on net care, such as frequency of washing and drying as revealed during pre-intervention interviews and surveys. These specific needs were included in a flip chart and as a consequence, knowledge on net washing and drying improved significantly in the intervention group (p < 0.001). Both qualitative and quantitative interviews showed that almost everyone knew that sleeping under a mosquito net would help prevent them from getting malaria. Overall, 93% of respondents had slept under a mosquito net the previous night. As per the World Malaria Report 2011 [1], 96% of those who possess a net actually sleep under it. Some of the reasons provided by some of Bhutaneses for not sleeping under a net, which were consistent with other studies [38], were that people do not sleep at home as they guard their crops during harvest seasons; additional nets have to be supplied to meet such practices [39,40].

IRS is a very effective tool for malaria prevention and Bhutan has used IRS since the inception of the malaria control initiatives in 1961, and is currently used in the community in combination with LLINs. While the benefit of IRS is evident, there is a paucity of evidence that such combination had any added cost-effective value in Bhutan’s malaria situation [41]. However, despite most households being sprayed with chemicals, many people did not know that their houses should not be plastered or whitewashed for at least six months after the spray to retain chemical effectiveness. As a result of inclusion in the educational sessions in the intervention group, the number of respondents who knew this improved significantly from 0.7% in pre-intervention to 93.5% during the post-intervention survey (p < 0.001) as compared to control group which increased from 1.5% in pre-intervention to 9.8% in post-intervention group (p = 0.004). This indicates that focused educational sessions makes a significant improvement if the baseline knowledge is very low. The main complaint of the community revealed during focus group discussion was that after the current IRS, there were more mosquitoes and bed bugs in households. This could be due to reasons that the programme had changed the IRS chemical from deltamethrin which is more toxic, to lambda cyphalothrin which is more insect deterrent [41]. Therefore, appropriate information has to be provided before any change in the interventions to help prevent misperceptions.

The findings of this study on KAP were similar to a malaria indicator survey conducted in Bhutan [36] and KAP studies conducted in Swaziland [42], in Malaysia [43] and in Vietnam [44] with the majority of respondents knowing the causes of malaria, their symptoms and seeking health facility as the first line of treatment for malaria. However, the community knowledge and practices on malaria prevention and control were better in this study as compared to studies conducted in Nepal [11] and in India [15]. This could be due to better health care delivery systems through the public health services and size of the population. As revealed both by quantitative and qualitative results, almost everyone had heard about malaria. Other studies [17,45,46] also showed that if health education was planned after proper need assessment, implemented and evaluated by involving community and community leaders throughout the process, it can show good results even in illiterate, rural communities. This study found the major knowledge improvement in topics that had very minimal baseline understanding, such as care of LLIN, plastering of houses after IRS, which could be due to this intervention being conducted after understanding the knowledge gap, community needs and incorporating the needs in the interventions to be implemented by CAGs [47,48]. Such community empowerment interventions can be used not only in malaria but could be of value across many public health issues, such as improving mother and child health, HIV/AIDS, etc. [23,49,50]. The training of the CAG members strengthened their interest for malaria prevention and control and even volunteered to carry out IRS activities within their communities, which was traditionally done by health workers.

## Conclusions

This study showed that by using CAGs for education, communities can improve KAP of malaria prevention and control. This interventional study was successful in enabling communities to learn, take initiatives and participate in malaria prevention and control in their locality and hence has potential and scope for expansion into other malaria-endemic areas of Bhutan. Further, CAGs can be utilized as the gateway for community development at the lowest administrative level by various sectors and agencies in developing the community. The methodology applied here can be utilized for the same purpose with other diseases, such as improving knowledge and awareness on HIV/AIDS, condom distribution, strengthening tuberculosis directly observed therapy programme in the community, promoting community referral systems, and in improving the sanitation and hygiene of the community. This study showed that CDI was effective for improving KAP on malaria prevention and control; however, the post-intervention effect was measured after six months of intervention. Further studies could be conducted to establish the long-term effect and the sustainability of CDI, within the context of malaria elimination in Bhutan. Research could be carried out to understand the uniqueness of these ethnic groups and the impact on malaria prevention and control to develop ethically inclusive interventions within the context of malaria elimination in Bhutan.

The limitations of the study were as follows:

1. This study was not a randomized control study. Hence malaria prevention and control activities including distribution of LLINs, awareness programmes by the district health and vector-borne disease control programme were provided as usual both in intervention and control areas. Further, there was no control over the socio-economic and demographic differences which could have affected the results.

2. The study period was rather short with only about six months gap after the intervention started until evaluation through the post-intervention survey. Therefore, the time duration was rather short to assess the real outcome of the intervention that is to see the change in the behaviour of the people.

3. The community leaders were all newly elected and showed lots of enthusiasm to improve their community and were susceptible to any new idea for the benefit of the community. This would pose a challenge for expansion of the intervention, if such supports were not rendered as their tenure matures.

4. The study coincided during monsoon season. Therefore, roadblocks due to heavy monsoon and swollen river belts were sometimes a hindrance to proper monitoring.

## Competing interests

The authors declare that they have no competing interests.

## Authors’ contributions

TT formulated and implemented, wrote and reviewed the manuscript. KN-B, CET, and UD formulated, supervised and reviewed the manuscript. DP and SPD collaborated and reviewed the manuscript. All authors read and approved the final manuscript.
